# PARENTAL STYLE MEDIATES GRANDCHILD DISTRESS: PARENTAL ROLE DEMANDS RELATIONSHIPS AMONG CUSTODIAL GRANDPARENTS

**DOI:** 10.1093/geroni/igae098.2731

**Published:** 2024-12-31

**Authors:** Bert Hayslip, Julian Montoro-Rodriguez, Janelle Fassi

**Affiliations:** University of North Texas, Denton, Texas, United States; University of North Carolina Charlotte, Charlotte, North Carolina, United States; University of Massachusetts Boston, Goffstown, New Hampshire, United States

## Abstract

While limited work has explored parenting practices among custodial grandparents, none has incorporated parental style, a construct proven valuable in predicting a variety of child outcomes of significance, in understanding parenting among custodial grandparents. This study evaluated the impact of grandchild distress (internalizing/externalizing behaviors) on Parental Efficacy, Parental Role Strain, and Parental Role Stress as mediated by parental styles (Laissez-Faire, Authoritative, Authoritarian) in a sample of 238 custodial grandparents (M age = 58.06). AMOS structural equation findings yielded a model which fit the data well (X2/df = 1.72, CFI = 0.939, TLI = 0.910, RMSE = 0.055). This model indicated that the Laissez-Faire and Authoritarian styles each predicted either Role Stress or Parental Efficacy. Laissez-Faire’s mediating role was driven to a minimal extent by grandchild internalizing/externalizing behaviors per se, while its mediating role was defined principally by grandparent age as it related to such behaviors in predicting Parental Efficacy (beta = -.54, p <.05). and Parental Role Stress (beta =.36, p <.05). The mediating role of the Authoritarian style was principally explained by child externalizing behaviors (beta =.22, p <.05) in predicting Parental Efficacy. Additionally, while the Authoritative style did not emerge as mediational in nature, both internalizing and externalizing behaviors had direct relationships (p <.05) to Role Stress and Role Strain. These finding illuminate the role of parental styles as key to understanding the parental challenges that grandparents face in managing the distress that their grandchildren are experiencing.

